# Supplementary material for: Impaired yolk sac NAD metabolism disrupts murine embryogenesis with relevance to human birth defects
Source: eLife. 2025 Mar 6;13:RP97649. doi: 10.7554/eLife.97649 (PMC11884786; doi:10.7554/eLife.97649)
Supplement: Supplementary file 1. [file elife-97649-supp1.docx]

Supplementary File 1. HAAO enzyme activity in embryonic liver, yolk sac, placenta, and embryo at different stages in gestation.

| **Tissue** | **Stage** | **#** | **Vol. buffer / mg weight** |  | **HAAO activity**  **(μmol min^-1^ mg protein^-1^)** | | | | | | | | **One-way ANOVA**  ***p*** |
| --- | --- | --- | --- | --- | --- | --- | --- | --- | --- | --- | --- | --- | --- |
|  |  |  |  |  |  |  |  |  |  |  |  |  |  |
|  |  |  |  |  | ***Haao+/+*** | **n** |  | ***Haao+/-*** | **n** |  | ***Haao-/-*** | **n** |  |
| Embryonic liver | E12.5 | 4-7 | 3 µL |  | 0.76 ± 0.04 | 4 |  | 0.62 ± 0.19 | 4 |  | 0.46 ± 0.07 | 3 | 0.0383 |
|  | E13.5 | 2-3 | 3 µL |  | 1.70 ± 0.54 | 4 |  | 1.49 ± 0.42 | 4 |  | 0.45 ± 0.08 | 4 | 0.0034 |
|  | E14.5 | 1 | 4 µL |  | 3.30 ± 0.17 | 4 |  | 2.15 ± 0.17 | 4 |  | 0.69 ± 0.25 | 4 | <0.0001 |
|  | E15.5 | 1 | 4 µL |  | 4.77 ± 0.68 | 4 |  | 2.99 ± 0.87 | 5 |  | 0.29 ± 0.13 | 4 | <0.0001 |
|  | E16.5 | 1 | 4 µL |  | 8.82 ± 2.03 | 4 |  | 4.66 ± 1.55 | 4 |  | 0.44 ± 0.04 | 4 | <0.0001 |
|  | E17.5 | 1 | 5 µL |  | 15.64 ± 4.21 | 4 |  | 8.45 ± 3.65 | 4 |  | 0.47 ± 0.06 | 4 | <0.0001 |
| Yolk sac | E11.5 | 2-3 | 3 µL |  | 10.73 ± 3.89 | 4 |  | 5.75 ± 1.39 | 4 |  | 0.12 ± 0.83 | 4 | 0.0006 |
|  | E12.5 | 1-2 | 3 µL |  | 10.68 ± 3.38 | 4 |  | 5.76 ± 2.20 | 4 |  | 0.35 ± 0.35 | 4 | 0.0005 |
|  | E13.5 | 1 | 3.5 µL |  | 11.35 ±2.89 | 4 |  | 5.95 ± 1.45 | 5 |  | 0.48 ± 0.14 | 4 | <0.0001 |
|  | E14.5 | 1 | 4 µL |  | 8.87 ± 2.49 | 4 |  | 4.22 ± 0.34 | 4 |  | 0.50 ± 0.22 | 4 | <0.0001 |
|  | E15.5 | 1 | 4 µL |  | 5.19 ± 0.70 | 4 |  | 2.91 ± 0.78 | 5 |  | 0.32 ± 0.19 | 4 | <0.0001 |
|  | E16.5 | 1 | 4 µL |  | 5.54 ± 1.03 | 4 |  | 2.64 ± 0.30 | 4 |  | 0.17 ± 0.15 | 4 | <0.0001 |
|  | E17.5 | 1 | 4 µL |  | 4.05 ± 0.46 | 4 |  | 2.26 ± 0.29 | 5 |  | 0.25 ± 0.17 | 4 | <0.0001 |
| Placenta | E11.5 | 1 | 3 µL |  | 0.49 ± 0.19 | 4 |  | 0.37 ± 0.02 | 4 |  | 0.49 ± 0.16 | 4 | 0.3952 |
|  | E12.5 | 1 | 4.5 µL |  | 0.32 ± 0.09 | 4 |  | 0.42 ± 0.09 | 4 |  | 0.44 ± 0.11 | 4 | 0.2221 |
|  | E13.5 | 1 | 4.5 µL |  | 0.43 ± 0.17 | 4 |  | 0.32 ± 0.12 | 4 |  | 0.44 ± 0.07 | 4 | 0.3593 |
|  | E14.5 | 1 | 4.5 µL |  | 0.42 ± 0.06 | 4 |  | 0.55 ± 0.14 | 4 |  | 0.44 ± 0.18 | 4 | 0.3907 |
|  | E15.5 | 1 | 5 µL |  | 0.40 ± 0.25 | 4 |  | 0.48 ± 0.23 | 4 |  | 0.49 ± 0.27 | 4 | 0.8401 |
|  | E16.5 | 1 | 5 µL |  | 0.50 ± 0.26 | 4 |  | 0.50 ± 0.10 | 4 |  | 0.53 ± 0.28 | 4 | 0.9739 |
|  | E17.5 | 1 | 5 µL |  | 0.44 ± 0.03 | 4 |  | 0.45 ± 0.19 | 4 |  | 0.53 ± 0.10 | 4 | 0.5795 |
| Embryo | E11.5 | 1 | 2.5 µL |  | 0.88 ± 0.34 | 4 |  | n.d. |  |  | n.d. |  |  |

E = embryonic day; # = number of samples pooled per biological replicate; n = number of biological replicates.

Data shown as mean ± standard deviation. Statistical significance was calculated by one-way ANOVA comparing the three *Haao* genotypes at each embryonic stage. n.d. = not determined.
