# Supplementary material for: Impaired yolk sac NAD metabolism disrupts murine embryogenesis with relevance to human birth defects
Source: eLife. 2025 Mar 6;13:RP97649. doi: 10.7554/eLife.97649 (PMC11884786; doi:10.7554/eLife.97649)
Supplement: Supplementary file 2. [file elife-97649-supp2.docx]

Supplementary File 2. NAD metabolite concentrations in embryonic liver at E14.5 by *Haao* genotype, as measured by UHPLC-MS/MS.

| **Metabolite** | ***Haao+/+*** | ***Haao+/−*** | ***Haao−/−*** | ***p^b^*** |  |
| --- | --- | --- | --- | --- | --- |
| TRP (nmol/g) | 77.3 ± 4.9 | 62.4 ± 34 | 81.7 ± 18.3 | 0.4932 |  |
| KYN (nmol/g) | 6.35 ± 0.58 | 5.11 ± 1.79 | 6.19 ± 2.14 | 0.6494 |  |
| 3HK (nmol/g) | 0.67 ± 0.08 | 0.66 ± 0.17 | 0.95 ± 0.17 | **0.0447** |  |
| 3HAA (nmol/g) | 0.11 ± 0.03 | 0.70 ± 0.97 | 5.09 ± 2.73 | **0.0141** |  |
| QA (nmol/g) | 2.25 ± 0.38 | 3.15 ± 0.98 | <LOD | n.a. |  |
| NAMN (pmol/g) | 33.0 ± 6.36 | 39.1 ± 27.1 | 6.12 ± 1.65 | 0.0951 |  |
| NAD+ (nmol/g) | 96.0 ± 4.2 | 81.2 ± 9.2 | 53.0 ± 7.6 | **0.0001** |  |
| NAM (nmol/g) | 20.9 ± 5.5 | 19.7 ± 7.3 | 39.8 ± 12.3 | **0.0325** |  |
| NMN (nmol/g) | 0.35 ± 0.06 | 0.31 ± 0.15 | 0.42 ± 0.10 | 0.3660 |  |
| 2PY (nmol/g) | 0.34 ± 0.11 | 0.16 ± 0.23 | 0.38 ± 0.20 | 0.3020 |  |
| 4PY (nmol/g) | 0.43 ± 0.16 | 0.20 ± 0.31 | 0.47 ± 0.25 | 0.2257 |  |
| KA (pmol/g) | 93.7 ± 26.7 | 78.2 ± 35.6 | 95.3 ± 26.3 | 0.7123 |  |
| KYN : TRP^a^ | 0.08 ± 0.02 | 0.09 ± 0.02 | 0.08 ± 0.02 | 0.6246 |  |

Metabolite concentrations are normalised to wet weight measured at dissection. TRP = L-tryptophan; KYN = L-kynurenine; 3HK = 3-hydroxykynurenine; 3HAA = 3-hydroxyanthranilic acid; QA = quinolinic acid; NAMN = nicotinic acid mononucleotide; NAD+ = nicotinamide adenine dinucleotide; NAM = nicotinamide; NMN = nicotinamide mononucleotide; 2PY = N-methyl-2-pyridone-5-carboxamide; 4PY = N-methyl-4-pyridone-5-carboxamide; KA = kynurenic acid. Number of biological replicates is n = 3 for *Haao^+/+^* and *Haao^+/-^*, n = 5 for *Haao^-/-^*. Data is shown as mean ± standard deviation. <LOD = below the detection limit (signal:noise <3). n.a. = not applicable (significance cannot be calculated).

^a^KYN:TRP ratio was calculated for individual samples, then summarised.

^b^Statistical significance was calculated by one-way ANOVA comparing the three *Haao* genotypes, with *p*<0.05 highlighted in bold.
