# Supplementary material for: Impaired yolk sac NAD metabolism disrupts murine embryogenesis with relevance to human birth defects
Source: eLife. 2025 Mar 6;13:RP97649. doi: 10.7554/eLife.97649 (PMC11884786; doi:10.7554/eLife.97649)
Supplement: Supplementary file 3. [file elife-97649-supp3.docx]

Supplementary File 3. NAD metabolite concentrations in the visceral yolk sac at E10.5, E12.5 and E14.5 by *Haao* genotype, as measured by UHPLC-MS/MS.

|  |  | **Yolk sac** | | | |  |
| --- | --- | --- | --- | --- | --- | --- |
| **Metabolite** | **Stage** | **+/+** | **+/−** | **−/−** | ***p^b^*** |  |
| TRP | E10.5 | 49.7 ± 9.5 | 61.7 ± 12.7 | 72.0 ± 17.8 | 0.1822 |  |
| (nmol/g) | E12.5 | 150 ± 42 | 133 ± 23 | 161.8 ± 35 | 0.5446 |  |
|  | E14.5 | 159 ± 14 | 149 ± 38 | 145 ± 33 | 0.8105 |  |
| KYN | E10.5 | 7.3 ± 2.0 | 9.3 ± 3.7 | 11.4 ± 2.8 | 0.2160 |  |
| (nmol/g) | E12.5 | 37.3 ± 6.1 | 43.4 ± 1.7 | 39.8 ± 5.8 | 0.3917 |  |
|  | E14.5 | 49.9 ± 7.0 | 62.6 ± 21.0 | 58.7 ± 9.7 | 0.5033 |  |
| 3HK | E10.5 | 0.26 ± 0.14 | 0.48 ± 0.27 | 0.83 ± 0.38 | 0.0839 |  |
| (nmol/g) | E12.5 | 1.07 ± 0.38 | 0.82 ± 0.42 | 3.30 ± 1.05 | **0.0039** |  |
|  | E14.5 | 0.74 ± 0.25 | 0.74 ± 0.28 | 0.84 ± 0.18 | 0.7414 |  |
| 3HAA | E10.5 | <LOD | <LOD | 6.2 ± 1.45 | n.a. |  |
| (nmol/g) | E12.5 | 0.40 ± 0.11 | 0.69 ± 0.03 | 12.5 ± 3.4 | **0.0022** |  |
|  | E14.5 | 0.21 ± 0.07 | 0.33 ± 0.09 | 5.19 ± 0.55 | **<0.0001** |  |
| NAMN | E10.5 | 0.20 ± 0.04 | 0.39 ± 0.22 | <LOD | n.a. |  |
| (nmol/g) | E12.5 | 0.80 ± 0.44 | 1.10 ± 0.65 | <LOD | n.a. |  |
|  | E14.5 | 0.50 ± 0.09 | 0.62 ± 0.22 | 0.04 ± 0.02 | **0.0003** |  |
| NAD+ | E10.5 | 48.7 ± 7.8 | 55.3 ± 14.3 | 50.5 ± 3.6 | 0.6304 |  |
| (nmol/g) | E12.5 | 116.6 ± 17 | 99.3 ± 17.6 | 92.1 ± 7.1 | 0.0933 |  |
|  | E14.5 | 82.9 ± 11.2 | 87.3 ± 28.1 | 83.2 ± 16.9 | 0.9501 |  |
| NAM | E10.5 | 15.2 ± 3.4 | 13.2 ± 5.1 | 7.8 ± 1.6 | **0.0303** |  |
| (nmol/g) | E12.5 | 17.2 ± 4.4 | 29.9 ± 29.5 | 19.2 ± 8.0 | 0.5815 |  |
|  | E14.5 | 14.0 ± 3.5 | 16.9 ± 3.1 | 15.5 ± 4.9 | 0.7124 |  |
| NMN | E10.5 | 0.28 ± 0.11 | 0.35 ± 0.18 | 0.34 ± 0.14 | 0.8232 |  |
| (nmol/g) | E12.5 | 2.65 ± 0.78 | 2.84 ± 1.73 | 2.33 ± 1.41 | 0.8728 |  |
|  | E14.5 | 3.12 ± 0.60 | 4.04 ± 0.69 | 4.25 ± 1.65 | 0.4826 |  |
| 2PY | E10.5 | 0.13 ± 0.03 | 0.12 ± 0.03 | 0.05 ± 0.04 | **0.0215** |  |
| (nmol/g) | E12.5 | 0.28 ± 0.05 | 0.17 ± 0.04 | 0.23 ± 0.09 | 0.2284 |  |
|  | E14.5 | 0.27 ± 0.09 | 0.22 ± 0.18 | 0.25 ± 0.13 | 0.9103 |  |
| 4PY | E10.5 | 0.16 ± 0.02 | 0.14 ± 0.03 | 0.07 ± 0.03 | **0.0083** |  |
| (nmol/g) | E12.5 | 0.48 ± 0.11 | 0.28 ± 0.08 | 0.41 ± 0.15 | 0.2062 |  |
|  | E14.5 | 0.80 ± 0.14 | 0.76 ± 0.61 | 0.75 ± 0.42 | 0.9879 |  |
| KA | E10.5 | 0.12 ± 0.18 | 0.11 ± 0.16 | 0.14 ± 0.38 | 0.4329 |  |
| (nmol/g) | E12.5 | 0.72 ± 0.18 | 0.80 ± 0.29 | 0.99 ± 0.29 | 0.3651 |  |
|  | E14.5 | 0.50 ± 0.12 | 0.56 ± 0.23 | 0.63 ± 0.26 | 0.6987 |  |
| KYN : TRP^a^ | E10.5 | 0.15 ± 0.04 | 0.15 ± 0.04 | 0.16 ± 0.04 | 0.8730 |  |
|  | E12.5 | 0.26 ± 0.04 | 0.33 ± 0.04 | 0.25 ± 0.05 | 0.1102 |  |
|  | E14.5 | 0.31 ± 0.03 | 0.42 ± 0.07 | 0.42 ± 0.08 | 0.1266 |  |

Metabolite concentrations are normalised to wet weight measured at dissection. TRP = L-tryptophan; KYN = L-kynurenine; 3HK = 3-hydroxykynurenine; 3HAA = 3-hydroxyanthranilic acid; NAMN = nicotinic acid mononucleotide; NAD+ = nicotinamide adenine dinucleotide; NAM = nicotinamide; NMN = nicotinamide mononucleotide; 2PY = N-methyl-2-pyridone-5-carboxamide; 4PY = N-methyl-4-pyridone-5-carboxamide; KA = kynurenic acid. Number of biological replicates is n = 3 for *Haao^+/+^* and *Haao^+/-^*, n = 5 for *Haao^-/-^*. Data is shown as mean ± standard deviation. <LOD = below the detection limit (signal:noise <3). n.a. = not applicable (significance cannot be calculated).

^a^KYN:TRP ratio was calculated for individual samples, then summarised.

^b^Statistical significance was calculated by one-way ANOVA comparing the three *Haao* genotypes, with *p*<0.05 highlighted in bold.
