# Supplementary material for: Impaired yolk sac NAD metabolism disrupts murine embryogenesis with relevance to human birth defects
Source: eLife. 2025 Mar 6;13:RP97649. doi: 10.7554/eLife.97649 (PMC11884786; doi:10.7554/eLife.97649)
Supplement: Supplementary file 4. [file elife-97649-supp4.docx]

Supplementary File 4. Summary of congenital malformation types and incidence in embryos at E18.5 arising from maternal NW3.5 diet provision between E7.5 and E10.5.

|  |  |  | **Embryonic genotype** | |
| --- | --- | --- | --- | --- |
| **Location** | **Malformation** |  | ***Haao*-/-** | ***Haao*+/-** |
| Heart | *Total^a^* |  | 8/30 (27.7%) | 1/20 (5%) |
|  | BAV |  | 5/30 (16.7%) | - |
|  | VSD |  | 1/30 (3.3%) | - |
|  | mVSD |  | - | 1/20 (5%) |
|  | Other structural abnormalities^b^ |  | 3/30 (10%) | - |
| Vertebrae and ribs^c^ | *Total* |  | 19/30 (63.3%) | 0/20 (0%) |
|  | Cervical vertebrae |  | 7/30 (23.3%) | - |
|  | Thoracic vertebrae |  | 8/30 (26.7%) | - |
|  | Lumbar vertebrae |  | 7/30 (23.3%) | - |
|  | Sacral vertebrae |  | 6/30 (20%) | - |
|  | Sternum |  | 4/30 (13.3%) | - |
|  | Underdeveloped / missing ribs |  | 13/30 (43.3%) | - |
| Kidneys^d^ | *Total* |  | 25/30 (83.3%) | 0/20 (0%) |
|  | Hypoplasia / agenesis |  | 25/30 (83.3%) | - |
|  | Dysmorphic |  | 1/30 (3.3%) | - |
| Tail | *Total* |  | 22/30 (73.3%) | 0/20 (0%) |
|  | Caudal agenesis |  | 22/30 (73.3%) | - |
| Digits and Limbs | *Total* |  | 12/30 (40%) | 0/20 (0%) |
|  | Preaxial polydactyly |  | 7/30 (23.3%) | - |
|  | Digit-like thumb |  | 3/30 (10%) | - |
|  | Syndactyly |  | 3/30 (10%) | - |
|  | Talipes |  | 1/30 (3.3%) | - |
| Eyes | *Total* |  | 8/30 (26.7%) | 0/20 (0%) |
|  | Microphthalmia |  | 8/30 (26.7%) | - |
| Abdominal wall | *Total* |  | 1/30 (3.3%) | 0/20 (0%) |
|  | Omphalocele |  | 1/30 (3.3%) | - |

BAV = Bicuspid aortic valve; VSD = ventricular septal defect; mVSD = muscular ventricular septal defect.

^a^Total refers to total number of embryos having a malformation in the indicated location. Note that embryos may have multiple malformation types in each location.

^b^Other structural abnormalities refers to compact myocardium in the right ventricle (observed in two embryos) and deflated atria (observed in one embryo).

^c^Vertebral defects were included or discounted using previously established criteria (Cuny et al., 2020). Observed and counted defects include vertebral fusions, butterfly vertebrae, and hemivertebrae. Sternum defects include reduced ossification of sternebrae and asymmetrical fusion of ribs to the sternum.

^d^Kidney defects were classified as described previously (Cuny et al., 2020). In brief, kidneys with a length (tip to tip) of ≤ 1.5 mm in length were counted as hypoplastic, because the average length of a normal kidney at E18.5 is 2.98 mm (2.75-3.375 mm). The kidney classified as dysmorphic had a cyst.

Cuny, H., Rapadas, M., Gereis, J., Martin, E., Kirk, R. B., Shi, H., & Dunwoodie, S. L. (2020). NAD deficiency due to environmental factors or gene-environment interactions causes congenital malformations and miscarriage in mice. *Proc Natl Acad Sci U S A*, *117*(7), 3738-3747. <https://doi.org/10.1073/pnas.1916588117>
