# Supplementary material for: Impaired yolk sac NAD metabolism disrupts murine embryogenesis with relevance to human birth defects
Source: eLife. 2025 Mar 6;13:RP97649. doi: 10.7554/eLife.97649 (PMC11884786; doi:10.7554/eLife.97649)
Supplement: Supplementary file 5. [file elife-97649-supp5.docx]

Supplementary File 5. NAD metabolite concentrations in the E10.5 embryo and yolk sac with maternal NW15 or NW3.5 diet provision, normalised by protein.

|  |  | **NW15** | | |  | **NW3.5** | | |  |  |
| --- | --- | --- | --- | --- | --- | --- | --- | --- | --- | --- |
| **Metabolite** | **Tissue** | ***Haao+/-*** | ***Haao−/−*** | ***p^a^*** |  | ***Haao+/−*** | ***Haao−/−*** | ***p^b^*** |  | ***p^c^*** |
| TRP  (nmol/mg) | Embryo | 2.52 ± 0.56 | 2.75 ± 0.88 | 0.9357 |  | 2.19 ± 0.88 | 2.04 ± 0.81 | 0.9839 |  | 0.2923 |
|  | Yolk sac | 2.90 ± 0.34 | 2.67 ± 0.30 | 0.4108 |  | 2.25 ± 0.14 | 2.38 ± 0.17 | 0.7999 |  | **0.0010** |
|  | Ratio | 0.90 ± 0.22 | 1.05 ± 0.30 |  |  | 0.99 ± 0.42 | 0.86 ± 0.35 |  |  |  |
| KYN  (nmol/g) | Embryo | 292 ± 71.7 | 317 ± 93.9 | 0.9657 |  | 245 ± 136 | 225 ± 117 | 0.9826 |  | 0.3170 |
|  | Yolk sac | 355 ± 83.1 | 337 ± 77.4 | 0.9593 |  | 263 ± 46.4 | 250 ± 30.8 | 0.9848 |  | **0.0194** |
|  | Ratio | 0.88 ± 0.23 | 1.02 ± 0.33 |  |  | 0.92 ± 0.46 | 0.89 ± 0.41 |  |  |  |
| 3HK  (nmol/g) | Embryo | 13.4 ± 5.2 | 13.3 ± 4.4 | >0.9999 |  | 11.9 ± 6.8 | 10.4 ± 5.9 | 0.9537 |  | 0.6762 |
|  | Yolk sac | 19.6 ± 4.3 | 30.2 ± 9.2 | 0.0299 |  | 13.2 ± 3.4 | 21.9 ± 5.5 | 0.0864 |  | **0.0009** |
|  | Ratio | 0.68 ± 0.20 | 0.44 ± 0.09 |  |  | 0.92 ± 0.53 | 0.51 ± 0.24 |  |  |  |
| 3HAA  (nmol/g) | Embryo | 34.3 ± 10.1 | 434 ± 109 | <0.0001 |  | 22.8 ± 13.8 | 297 ± 144 | **<0.0001** |  | **<0.0001** |
|  | Yolk sac | 42.3 ± 8.4 | 222 ± 25.5 | <0.0001 |  | 21.9 ± 5.2 | 179 ± 23.4 | **<0.0001** |  | **<0.0001** |
|  | Ratio | 0.82 ± 0.16 | 1.97 ± 0.50 |  |  | 1.04 ± 0.60 | 1.68 ± 0.75 |  |  |  |
| QA  (nmol/g) | Embryo | 164 ± 98.3 | <LOD | n.a. |  | 170 ± 79.0 | <LOD | n.a. |  | n.a. |
|  | Yolk sac | 225 ± 33.1 | <LOD | n.a. |  | 153 ± 32.9 | <LOD | n.a. |  | n.a. |
|  | Ratio | 0.75 ± 0.40 | n.a. |  |  | 1.06 ± 0.47 | n.a. |  |  |  |
| NAMN  (nmol/g) | Embryo | 0.100 ± 0.302 | <LOD | n.a. |  | 0.078 ± 0.019 | 0.066 ± 0.017 | 0.7229 |  | 0.0704 |
|  | Yolk sac | 2.14 ± 0.33 | <LOD | n.a. |  | 1.24 ± 0.23 | <LOD | n.a. |  | n.a. |
|  | Ratio | 0.05 ± 0.02 | n.a. |  |  | 0.06 ± 0.03 | n.a. |  |  |  |
| NAAD  (nmol/g) | Embryo | 1.99 ± 1.42 | 0.085 ± 0.040 | 0.0006 |  | 0.847 ± 0.741 | 0.032 ± 0.019 | 0.2912 |  | **0.0003** |
|  | Yolk sac | 12.3 ± 5.6 | 0.334 ± 0.340 | <0.0001 |  | 5.94 ± 1.07 | 0.100 ± 0.115 | **0.0420** |  | **<0.0001** |
|  | Ratio | 0.18 ± 0.12 | 0.46 ± 0.36 |  |  | 0.15 ± 0.14 | 1.13 ± 1.32 |  |  |  |
| NAD+  (nmol/mg) | Embryo | 4.73 ± 1.42 | 3.22 ± 0.85 | 0.0121 |  | 2.57 ± 0.62 | 1.12 ± 0.33 | **0.0164** |  | **<0.0001** |
|  | Yolk sac | 4.97 ± 0.57 | 3.54 ± 0.45 | <0.0001 |  | 4.44 ± 0.30 | 2.42 ± 0.41 | **<0.0001** |  | **<0.0001** |
|  | Ratio | 0.98 ± 0.34 | 0.92 ± 0.31 |  |  | 0.58 ± 0.12 | 0.47 ± 0.19 |  |  |  |
| NAM  (nmol/g) | Embryo | 145 ± 40.2 | 89.8 ± 17.1 | 0.0016 |  | 105 ± 30.0 | 55.0 ± 8.5 | **0.0047** |  | **<0.0001** |
|  | Yolk sac | 605 ± 129 | 465 ± 54.9 | 0.0265 |  | 519 ± 35.0 | 359 ± 60.8 | **0.0103** |  | **0.0002** |
|  | Ratio | 0.24 ± 0.07 | 0.20 ± 0.04 |  |  | 0.20 ± 0.05 | 0.16 ± 0.03 |  |  |  |
| NMN  (nmol/g) | Embryo | 13.3 ± 3.5 | 9.30 ± 2.04 | 0.0115 |  | 12.2 ± 1.6 | 8.56 ± 1.93 | **0.0221** |  | **0.0009** |
|  | Yolk sac | 23.1 ± 5.8 | 13.2 ± 2.2 | 0.0022 |  | 14.9 ± 4.9 | 9.42 ± 1.97 | 0.1298 |  | **<0.0001** |
|  | Ratio | 0.64 ± 0.26 | 0.70 ± 0.22 |  |  | 0.88 ± 0.28 | 0.93 ± 0.27 |  |  |  |
| 2PY  (nmol/g) | Embryo | 1.33 ± 0.78 | 1.24 ± 0.58 | 0.9474 |  | 0.200 ± 0.085 | 0.218 ± 0.114 | >0.9999 |  | **<0.0001** |
|  | Yolk sac | <LOD | <LOD | n.d. |  | <LOD | <LOD | n.a. |  | n.a. |
|  | Ratio | n.a. | n.a. |  |  | n.a. | n.a. |  |  |  |
| 4PY  (nmol/g) | Embryo | 1.31 ± 0.78 | 1.46 ± 0.76 | 0.9474 |  | 0.192 ± 0.091 | 0.185 ± 0.092 | >0.9999 |  | **<0.0001** |
|  | Yolk sac | <LOD | <LOD | n.d. |  | <LOD | <LOD | n.d. |  | n.d. |
|  | Ratio | n.a. | n.a. |  |  | n.a. | n.a. |  |  |  |
| KA  (nmol/g) | Embryo | 1.68 ± 0.53 | 1.70 ± 0.48 | 0.9998 |  | 1.58 ± 0.55 | 1.45 ± 0.45 | 0.9552 |  | 0.7401 |
|  | Yolk sac | 2.40 ± 0.53 | 2.55 ± 0.52 | 0.9355 |  | 2.06 ± 0.29 | 1.97 ± 0.43 | 0.9864 |  | 0.2504 |
|  | Ratio | 0.70 ± 0.23 | 0.68 ± 0.18 |  |  | 0.80 ± 0.33 | 0.77 ± 0.20 |  |  |  |

Metabolite concentrations are normalised to total protein content in the solid fraction after metabolite extraction (tissue pellet). TRP = L-tryptophan; KYN = L-kynurenine; 3HK = 3-hydroxykynurenine; 3HAA = 3-hydroxyanthranilic acid; QA = quinolinic acid; NAMN = nicotinic acid mononucleotide; NAAD = nicotinic acid adenine dinucleotide; NAD+ = nicotinamide adenine dinucleotide; NAM = nicotinamide; NMN = nicotinamide mononucleotide; 2PY = N-methyl-2-pyridone-5-carboxamide; 4PY = N-methyl-4-pyridone-5-carboxamide; KA = kynurenic acid. n = 6 for yolk sacs, n = 8 for embryos per *Haao* genotype per condition; data shown as mean ± standard deviation. <LOD = below the detection limit (signal:noise <3). n.a. = not applicable (ratio or significance cannot be calculated).

^a,b^Statistical significance was calculated by one-way ANOVA with Tukey’s multiple comparisons test comparing the two *Haao* genotypes and two dietary treatments, with the *p* values of comparing *Haao*^+/-^ and *Haao*^-/-^ on NW15 (a) and *Haao*^+/-^ and *Haao*^-/-^ on NW3.5 (b) shown and *p*<0.05 highlighted in bold.

^c^Statistical significance was calculated by one-way ANOVA comparing all four groups.
