# Supplementary material for: Impaired yolk sac NAD metabolism disrupts murine embryogenesis with relevance to human birth defects
Source: eLife. 2025 Mar 6;13:RP97649. doi: 10.7554/eLife.97649 (PMC11884786; doi:10.7554/eLife.97649)
Supplement: Supplementary file 6. [file elife-97649-supp6.docx]

Supplementary File 6. NAD metabolite concentrations in the E10.5 embryo and yolk sac with maternal NW15 or NW3.5 diet provision, normalised by wet weight.

|  |  | **NW15** | | |  | **NW3.5** | | |  |  |
| --- | --- | --- | --- | --- | --- | --- | --- | --- | --- | --- |
| **Metabolite** | **Tissue** | ***Haao+/-*** | ***Haao−/−*** | ***P ^a^*** |  | ***Haao+/−*** | ***Haao−/−*** | ***p^b^*** |  | ***p^c^*** |
| TRP  (nmol/g) | Embryo | 65.8 ± 13.1 | 72.7 ± 23.4 | 0.9167 |  | 61.5 ± 25.2 | 57.6 ± 21.8 | 0.9825 |  | 0.5388 |
|  | Yolk sac | 118 ± 14.3 | 118 ± 11.8 | 0.9997 |  | 108 ± 5.4 | 114 ± 7.3 | 0.6940 |  | 0.2772 |
|  | Ratio | 0.57 ± 0.13 | 0.62 ± 0.18 |  |  | 0.57 ± 0.24 | 0.52 ± 0.21 |  |  |  |
| KYN  (nmol/g) | Embryo | 7.55 ± 1.48 | 8.33 ± 2.37 | 0.9430 |  | 6.77 ± 3.65 | 6.28 ± 3.11 | 0.9851 |  | 0.4833 |
|  | Yolk sac | 14.3 ± 2.4 | 15.1 ± 3.9 | 0.9610 |  | 12.7 ± 2.6 | 12.1 ± 1.7 | 0.9794 |  | 0.1914 |
|  | Ratio | 0.56 ± 0.11 | 0.60 ± 0.19 |  |  | 0.52 ± 0.24 | 0.54 ± 0.23 |  |  |  |
| 3HK  (nmol/g) | Embryo | 0.345 ± 0.120 | 0.353 ± 0.118 | 0.9996 |  | 0.329 ± 0.182 | 0.290 ± 0.158 | 0.9525 |  | 0.8353 |
|  | Yolk sac | 0.793 ± 0.133 | 1.363 ± 0.472 | 0.0198 |  | 0.638 ± 0.188 | 1.067 ± 0.309 | 0.1024 |  | **0.0029** |
|  | Ratio | 0.43 ± 0.12 | 0.26 ± 0.06 |  |  | 0.52 ± 0.29 | 0.31 ± 0.14 |  |  |  |
| 3HAA  (nmol/g) | Embryo | 0.907 ± 0.293 | 11.49 ± 3.18 | <0.0001 |  | 0.628 ± 0.364 | 8.30 ± 3.79 | **<0.0001** |  | **<0.0001** |
|  | Yolk sac | 1.72 ± 0.33 | 9.95 ± 1.51 | <0.0001 |  | 1.06 ± 0.30 | 8.68 ± 1.48 | **<0.0001** |  | **<0.0001** |
|  | Ratio | 0.52 ± 0.11 | 1.17 ± 0.30 |  |  | 0.58 ± 0.32 | 1.02 ± 0.43 |  |  |  |
| QA  (nmol/g) | Embryo | 4.19 ± 2.40 | <LOD | n.a. |  | 4.72 ± 2.12 | <LOD | n.a. |  | n.a. |
|  | Yolk sac | 9.12 ± 0.99 | <LOD | n.a. |  | 7.43 ± 1.83 | <LOD | n.a. |  | n.a. |
|  | Ratio | 0.47 ± 0.26 | n.a. |  |  | 0.60 ± 0.25 | n.a. |  |  |  |
| NAMN  (pmol/g) | Embryo | 2.63 ± 0.87 | <LOD | n.a. |  | 2.14 ± 0.49 | 1.93 ± 0.57 | 0.8607 |  | 0.1844 |
|  | Yolk sac | 87.3 ± 11.9 | <LOD | n.a. |  | 59.62 ± 12.40 | <LOD | n.a. |  | n.a. |
|  | Ratio | 0.60 ± 0.39 | n.a. |  |  | 0.37 ± 0.30 | n.a. |  |  |  |
| NAAD  (pmol/g) | Embryo | 50.9 ± 35.0 | 2.26 ± 1.09 | 0.0005 |  | 23.3 ± 20.2 | 0.910 ± 0.597 | 0.2217 |  | **0.0002** |
|  | Yolk sac | 491 ± 187 | 15.5 ± 16.0 | <0.0001 |  | 286 ± 56.2 | 4.89 ± 5.78 | **0.0038** |  | **<0.0001** |
|  | Ratio | 0.11 ± 0.07 | 0.28 ± 0.23 |  |  | 0.08 ± 0.08 | 0.68 ± 0.80 |  |  |  |
| NAD+  (nmol/g) | Embryo | 122.3 ± 31.1 | 85.6 ± 25.9 | 0.0143 |  | 72.0 ± 16.7 | 31.9 ± 10.1 | **0.0069** |  | **<0.0001** |
|  | Yolk sac | 203 ± 19.5 | 158 ± 17.2 | 0.0003 |  | 213 ± 10.3 | 116 ± 11.3 | **<0.0001** |  | **<0.0001** |
|  | Ratio | 0.62 ± 0.20 | 0.54 ± 0.18 |  |  | 0.33 ± 0.07 | 0.28 ± 0.10 |  |  |  |
| NAM  (nmol/g) | Embryo | 3.80 ± 0.98 | 2.38 ± 0.55 | 0.0024 |  | 2.94 ± 0.81 | 1.57 ± 0.30 | **0.0032** |  | **<0.0001** |
|  | Yolk sac | 24.6 ± 5.2 | 20.8 ± 3.1 | 0.3310 |  | 25.0 ± 2.1 | 17.4 ± 4.2 | **0.0144** |  | **0.0085** |
|  | Ratio | 0.15 ± 0.04 | 0.12 ± 0.03 |  |  | 0.12 ± 0.03 | 0.10 ± 0.02 |  |  |  |
| NMN  (nmol/g) | Embryo | 0.350 ± 0.094 | 0.246 ± 0.062 | 0.0456 |  | 0.346 ± 0.069 | 0.246 ± 0.069 | 0.0543 |  | **0.0068** |
|  | Yolk sac | 0.937 ± 0.210 | 0.592 ± 0.118 | 0.0165 |  | 0.717 ± 0.240 | 0.456 ± 0.119 | 0.0900 |  | **0.0013** |
|  | Ratio | 0.41 ± 0.16 | 0.42 ± 0.15 |  |  | 0.52 ± 0.21 | 0.56 ± 0.17 |  |  |  |
| 2PY  (pmol/g) | Embryo | 34.0 ± 18.9 | 33.4 ± 17.5 | 0.9997 |  | 5.67 ± 2.44 | 6.26 ± 3.35 | 0.9997 |  | **<0.0001** |
|  | Yolk sac | <LOD | <LOD | n.a. |  | <LOD | <LOD | n.a. |  | n.a. |
|  | Ratio | n.a. | n.a. |  |  | n.a. | n.a. |  |  |  |
| 4PY  (pmol/g) | Embryo | 33.7 ± 18.9 | 39.3 ± 22.7 | 0.8738 |  | 5.44 ± 2.64 | 5.30 ± 2.64 | >0.9999 |  | **<0.0001** |
|  | Yolk sac | <LOD | <LOD | n.a. |  | <LOD | <LOD | n.a. |  | n.a. |
|  | Ratio | n.a. | n.a. |  |  | n.a. | n.a. |  |  |  |
| KA  (pmol/g) | Embryo | 43.3 ± 11.8 | 44.7 ± 12.3 | 0.9960 |  | 43.9 ± 14.5 | 40.7 ± 11.4 | 0.9560 |  | 0.9278 |
|  | Yolk sac | 97.3 ± 19.5 | 115 ± 27.6 | 0.5470 |  | 98.9 ± 14.6 | 95.5 ± 24.6 | 0.9936 |  | 0.4428 |
|  | Ratio | 0.44 ± 0.14 | 0.40 ± 0.10 |  |  | 0.45 ± 0.16 | 0.47 ± 0.13 |  |  |  |

Metabolite concentrations are normalised to wet weight measured at dissection. TRP = L-tryptophan; KYN = L-kynurenine; 3HK = 3-hydroxykynurenine; 3HAA = 3-hydroxyanthranilic acid; QA = quinolinic acid; NAMN = nicotinic acid mononucleotide, NAAD = nicotinic acid adenine dinucleotide; NAD+, nicotinamide adenine dinucleotide; NAM = nicotinamide; NMN = nicotinamide mononucleotide; 2PY = N-methyl-2-pyridone-5-carboxamide; 4PY = N-methyl-4-pyridone-5-carboxamide; KA = kynurenic acid. n = 6 for yolk sacs, n = 8 for embryos per *Haao* genotype per condition; data shown as mean ± standard deviation. <LOD = below the detection limit (signal:noise <3). n.a. = not applicable (ratio or significance cannot be calculated).

^a,b^Statistical significance was calculated by one-way ANOVA with Tukey’s multiple comparisons test comparing the two *Haao* genotypes and two dietary treatments, with the *p* values of comparing *Haao*+/- and *Haao*^-/-^ on NW15 (a) and *Haao*^+/-^ and *Haao*^-/-^ on NW3.5 (b) shown and *p*<0.05 highlighted in bold.

^c^Statistical significance was calculated by one-way ANOVA comparing all four groups.
