# Supplementary material for: Impaired yolk sac NAD metabolism disrupts murine embryogenesis with relevance to human birth defects
Source: eLife. 2025 Mar 6;13:RP97649. doi: 10.7554/eLife.97649 (PMC11884786; doi:10.7554/eLife.97649)
Supplement: Supplementary file 7. [file elife-97649-supp7.docx]

**Supplementary File 7.** Primers for RT-qPCR.

| **Gene Symbol** | **Forward primer (5’-3’)** | **Reverse primer (5’-3’)** |
| --- | --- | --- |
| *Tdo2* | AGGTGCTGCTCTGCTTGTTT | TGAGCGTGTCAATGTCCATAA |
| *Ido2* | TGCCCTCAGACTTCCTCACT | CGCTGCTCACGGTAACTCTT |
| *Afmid* | AGCCACCTCCCAGAATGAC | TGGAACCACATCCAAGTGTC |
| *Kmo* | GAGCATTAACTTGGCCCTTTC | AGTGGATCATTCTGGCTTTCA |
| *Kynu* | GTTCAGTGGGCTGCACTTTT | CCCAGTCATGTAAGCGGAGT |
| *Haao* | ACAATGGGAGGGCAGTGTAT | CTTCTTACGGGCAGGGTCTT |
| *Qprt* | ACTGGTGGAGAAGTATGGGC | GGCTGCTACATTCCACCTCT |
| *Nadsyn1* | GCCAAAGGCAAAGGTGCAAG | TAGCGAACATTCCGGTGCAT |
| *Nmnat1* | GTGCCCAACTTGTGGAAGAT | CAGCACATCGGACTCGTAGA |
| *Nmnat3* | CCAGAGACCACCTACACCAAA | CCACCCGAATCCAGTCAG |
| *Ubc^a^* | CCCAGTGTTACCACCAAG | ATCACACCCAAGAACAAGC |
| *Ywhaz^b^* | CAGTAGATGGAGAAAGATTTGC | GGGACAATTAGGGAAGTAAGT |

^a^Previously published in Gu et al. (Gu et al., 2014). ^b^Previously published in Jeong et al. (Jeong et al., 2014).

Gu, Y., Shen, X., Zhou, D., Wang, Z., Zhang, N., Shan, Z., Jin, L., & Lei, L. (2014). Selection and Expression Profiles of Reference Genes in Mouse Preimplantation Embryos of Different Ploidies at Various Developmental Stages. *PLOS ONE*, *9*(6), e98956. <https://doi.org/10.1371/journal.pone.0098956>

Jeong, J.-K., Kang, M.-H., Gurunathan, S., Cho, S.-G., Park, C., Seo, H. G., & Kim, J.-H. (2014). Evaluation of reference genes in mouse preimplantation embryos for gene expression studies using real-time quantitative RT-PCR (RT-qPCR). *BMC Research Notes*, *7*(1), 675. <https://doi.org/10.1186/1756-0500-7-675>
