# Supplementary material for: Impaired yolk sac NAD metabolism disrupts murine embryogenesis with relevance to human birth defects
Source: eLife. 2025 Mar 6;13:RP97649. doi: 10.7554/eLife.97649 (PMC11884786; doi:10.7554/eLife.97649)
Supplement: Supplementary file 8. [file elife-97649-supp8.docx]

**Supplementary File 8.** Summary of published RNA-seq datasets used and assessed in this study.

| **Tissue** | **Species** | **Stage** | **Paper** | **Data type; url/ accession ID; subsetting** |
| --- | --- | --- | --- | --- |
| Liver | Mouse | E9.5-17.5 | (Mu et al., 2020) | scRNA-seq;  <https://db.cngb.org/search/project/CNP0000236/> ;  Subsetted out liver primordium, liver bud and hepatocytes |
| Placenta | Mouse | E9.5, E10.5, E12.5, E14.5 | (Marsh & Blelloch, 2020) | scRNA-seq;  <https://figshare.com/projects/Single_nuclei_RNA-seq_of_mouse_placental_labyrinth_development/92354> |
| Yolk sac | Mouse | E9.5, E10.5 | (Zhao & Choi, 2019) | scRNA-seq;  <https://www.ncbi.nlm.nih.gov/geo/query/acc.cgi?acc=GSM3732840> |
| Gastrulation atlas | Mouse | E6.5-8.5 | (Pijuan-Sala et al., 2019) | scRNA-seq; <https://bioconductor.org/packages/devel/data/experiment/vignettes/MouseGastrulationData/inst/doc/MouseGastrulationData.html> ;  Seurat objects were generated by loading one “sample” per embryonic stage. |
| Yolk sac | Human | ? | (Cindrova-Davies et al., 2017) | Bulk RNA-Seq;  <https://www.ebi.ac.uk/ena/browser/view/PRJEB18767> |
| Yolk sac | Human | 3-8 PCW | (Goh et al., 2023) | scRNA-Seq;  <https://app.cellatlas.io/yolk-sac/dataset/2/scatterplot> ;  Subsetted yolk sac endoderm |
| Liver | Human | Embryonic and adult | (Wesley et al., 2022) | scRNA-Seq;  <https://app.cellatlas.io/liver-development/dataset/7/scatterplot> |
| Organogenesis atlas | Mouse | E10.5 | (Cao et al., 2019) | scRNA-seq;  <https://oncoscape.v3.sttrcancer.org/atlas.gs.washington.edu.mouse.rna/downloads> ;  Subsetted E10.5 only |

Cao, J., Spielmann, M., Qiu, X., Huang, X., Ibrahim, D. M., Hill, A. J., Zhang, F., Mundlos, S., Christiansen, L., Steemers, F. J., Trapnell, C., & Shendure, J. (2019). The single-cell transcriptional landscape of mammalian organogenesis. *Nature*, *566*(7745), 496-502. <https://doi.org/10.1038/s41586-019-0969-x>

Cindrova-Davies, T., Jauniaux, E., Elliot, M. G., Gong, S., Burton, G. J., & Charnock-Jones, D. S. (2017). RNA-seq reveals conservation of function among the yolk sacs of human, mouse, and chicken. *Proceedings of the National Academy of Sciences*, *114*(24), E4753-E4761. <https://doi.org/doi:10.1073/pnas.1702560114>

Goh, I., Botting, R. A., Rose, A., Webb, S., Engelbert, J., Gitton, Y., Stephenson, E., Quiroga Londoño, M., Mather, M., Mende, N., Imaz-Rosshandler, I., Yang, L., Horsfall, D., Basurto-Lozada, D., Chipampe, N. J., Rook, V., Lee, J. T. H., Ton, M. L., Keitley, D., . . . Haniffa, M. (2023). Yolk sac cell atlas reveals multiorgan functions during human early development. *Science*, *381*(6659), eadd7564. <https://doi.org/10.1126/science.add7564>

Marsh, B., & Blelloch, R. (2020). Single nuclei RNA-seq of mouse placental labyrinth development. *eLife*, *9*, e60266. <https://doi.org/10.7554/eLife.60266>

Mu, T., Xu, L., Zhong, Y., Liu, X., Zhao, Z., Huang, C., Lan, X., Lufei, C., Zhou, Y., Su, Y., Xu, L., Jiang, M., Zhou, H., Lin, X., Wu, L., Peng, S., Liu, S., Brix, S., Dean, M., . . . Hou, Y. (2020). Embryonic liver developmental trajectory revealed by single-cell RNA sequencing in the Foxa2eGFP mouse. *Communications Biology*, *3*(1), 642. <https://doi.org/10.1038/s42003-020-01364-8>

Pijuan-Sala, B., Griffiths, J. A., Guibentif, C., Hiscock, T. W., Jawaid, W., Calero-Nieto, F. J., Mulas, C., Ibarra-Soria, X., Tyser, R. C. V., Ho, D. L. L., Reik, W., Srinivas, S., Simons, B. D., Nichols, J., Marioni, J. C., & Göttgens, B. (2019). A single-cell molecular map of mouse gastrulation and early organogenesis. *Nature*, *566*(7745), 490-495. <https://doi.org/10.1038/s41586-019-0933-9>

Wesley, B. T., Ross, A. D. B., Muraro, D., Miao, Z., Saxton, S., Tomaz, R. A., Morell, C. M., Ridley, K., Zacharis, E. D., Petrus-Reurer, S., Kraiczy, J., Mahbubani, K. T., Brown, S., Garcia-Bernardo, J., Alsinet, C., Gaffney, D., Horsfall, D., Tysoe, O. C., Botting, R. A., . . . Vallier, L. (2022). Single-cell atlas of human liver development reveals pathways directing hepatic cell fates. *Nat Cell Biol*, *24*(10), 1487-1498. <https://doi.org/10.1038/s41556-022-00989-7>

Zhao, H., & Choi, K. (2019). Single cell transcriptome dynamics from pluripotency to FLK1(+) mesoderm. *Development*, *146*(23). <https://doi.org/10.1242/dev.182097>
